# *Toxoplasma gondii* Seropositivity Interacts with Catechol-*O*-methyltransferase Val105/158Met Variation Increasing the Risk of Schizophrenia

**DOI:** 10.3390/genes13061088

**Published:** 2022-06-18

**Authors:** Paula Rovira, Blanca Gutiérrez, Antonio Sorlózano-Puerto, José Gutiérrez-Fernández, Esther Molina, Margarita Rivera, Rafael Martínez-Leal, Inmaculada Ibanez-Casas, María Victoria Martín-Laguna, Araceli Rosa, Francisco Torres-González, Jorge A. Cervilla

**Affiliations:** 1 Instituto de Neurociencias, Centro de Investigación Biomédica (CIBM), Universidad de Granada, 18016 Granada, Spain; inv.paularovira@ugr.es (P.R.); mrivera@ugr.es (M.R.); vivi_martin_laguna@hotmail.com (M.V.M.-L.); jcervilla@ugr.es (J.A.C.); 2 Departamento de Psiquiatría, Facultad de Medicina, Universidad de Granada, 18016 Granada, Spain; ftorres@ugr.es; 3 Instituto de Investigación Biosanitaria ibs.Granada, 18012 Granada, Spain; asp@ugr.es (A.S.-P.); josegf@ugr.es (J.G.-F.); 4 Vicerectorat de Recerca, Investigadora postdoctoral Margarita Salas, Universitat de Barcelona, 08007 Barcelona, Spain; 5 Departamento de Microbiología, Facultad de Medicina, Universidad de Granada, 18016 Granada, Spain; 6 Departamento de Enfermería, Facultad de Ciencias de la Salud, Universidad de Granada, 18071 Granada, Spain; 7 Departamento de Bioquímica y Biología Molecular II, Facultad de Farmacia, Universidad de Granada, 18071 Granada, Spain; 8 Unidad de Investigación en Discapacidad Intelectual y Trastornos del Desarrollo (UNIVIDD), Fundació Villablanca, IISPV, Departamento de Psicología, Universitat Rovira i Virgili, Centro de Investigación Biomédica en Red de Salud Mental (CIBERSAM), 43007 Reus, Spain; rafael.martinez@urv.cat; 9 Department of Psychology, State University of New York at Plattsburgh, Plattsburgh, 12901 NY, USA; iiban001@plattsburgh.edu; 10 Secció de Zoologia i Antropologia Biològica, Departament de Biologia Evolutiva, Ecologia i Ciències Ambientals, Facultat de Biologia, Institut de Biomedicina de la Universitat de Barcelona (IBUB), Universitat de Barcelona, Centro de Investigación Biomédica en Red de Salud Mental, Instituto de Salud Carlos III, 08028 Barcelona, Spain; araceli.rosa@ub.edu

**Keywords:** *COMT*, *Toxoplasma gondii*, gene–environment interaction, schizophrenia, infectious agents, case–control study

## Abstract

Schizophrenia is a heterogeneous and severe psychotic disorder. Epidemiological findings have suggested that the exposure to infectious agents such as *Toxoplasma gondii* (*T. gondii*) is associated with an increased risk for schizophrenia. On the other hand, there is evidence involving the catechol-O-methyltransferase (*COMT*) Val105/158Met polymorphism in the aetiology of schizophrenia since it alters the dopamine metabolism. A case–control study of 141 patients and 142 controls was conducted to analyse the polymorphism, the prevalence of anti-*T. gondii* IgG, and their interaction on the risk for schizophrenia. IgG were detected by ELISA, and genotyping was performed with TaqMan Real-Time PCR. Although no association was found between any *COMT* genotype and schizophrenia, we found a significant association between *T. gondii* seropositivity and the disorder (χ^2^ = 11.71; *p*-value < 0.001). Furthermore, the risk for schizophrenia conferred by *T. gondii* was modified by the *COMT* genotype, with those who had been exposed to the infection showing a different risk compared to that of nonexposed ones depending on the *COMT* genotype (χ^2^ for the interaction = 7.28, *p*-value = 0.007). This study provides evidence that the *COMT* genotype modifies the risk for schizophrenia conferred by *T. gondii* infection, with it being higher in those individuals with the Met/Met phenotype, intermediate in heterozygous, and lower in those with the Val/Val phenotype.

## 1. Introduction

Schizophrenia is a debilitating chronic psychiatric illness characterized by changes in perception, thought, affectivity, and behaviour. The search for mechanisms that underlie its aetiology seems to point towards interacting effects of neurochemical, genetic, and environmental risk factors [1]. Such interaction seems to result in alterations in dopaminergic neurotransmission, leading to psychotic symptoms [2].

Although disrupted neuronal function in schizophrenia likely extends beyond the synapse, the results of the largest genome-wide association study (GWAS) of schizophrenia performed so far [3] again indicated the pathophysiological importance of genes participating in synaptic transmission that are located in pre- and postsynaptic terminals. According to this, genetic variation in catechol-O-methyltransferase (*COMT*) continues to be of special interest since it is the principal metaboliser of dopamine in the synapse [4]. In this context, the most common functional polymorphism of *COMT* (named rs4680 (replacement of G472A) or Val105/158Met) has been associated with various cognitive phenotypes, changes in brain activation and structure, and psychiatric disorders. The replacement of G/A leads to the replacement of valine (Val) with methionine (Met) in codon 158 for membrane-bound COMT, and in codon 108 for soluble COMT. Thus, it has functional effects on enzyme activity, with Val/Val individuals having the highest activity of the protein, Val/Met carriers having intermediate activity, and those carrying Met/Met phenotype having the lowest activity [5]. This could translate into different degrees of vulnerability to schizophrenia, where a general nonspecific dysfunction of the dopaminergic neurotransmission pathway is widely accepted [2].

Despite the high heritability of schizophrenia, it is well-recognized that nongenetic factors such as certain neurotropic infections and other early life stressors can also play a role [6,7,8,9]. Maternal exposure to the influenza virus during pregnancy could increase the risk for schizophrenia in offspring, and other neurotropic viruses have been assessed as infectious agents increasing the risk for schizophrenia [10]. Similarly, other infectious agents such as protozoa are also potential risk factors. Due to its well-known neurotropism and the consequent changes in the human brain, *T.gondii* has been an agent of particularly great interest in psychiatry during the last few decades. Human acute infection with *T. gondii* can produce psychotic symptoms similar to those displayed by individuals with schizophrenia [11]. In fact, the latest meta-analysis of studies investigating anti-*T. gondii* IgG seropositivity as a measure of previous infection stated a higher prevalence of IgG in individuals with schizophrenia compared to controls [12]. *T. gondii* infection seems to alter the host’s ability to metabolize dopamine as a means to provoke behavioural changes in the host, leading to the completion of the parasite’s transmission cycle [13]. For this reason, we decided to determine anti-*T. gondii* IgG seropositivity in our sample. Provided that COMT functionality is chiefly influential to dopamine’s metabolism too, we set to test the plausible hypothesis that both *T. gondii* infection and the *COMT* genotype exert their risk for schizophrenia by interacting with each other via the modification of dopamine metabolism. In particular, we wanted to assess whether the risk for schizophrenia conferred by *T. gondii* infection is modified by the *COMT* Val105/158Met genetic variation.

## 2. Materials and Methods

### 2.1. Sample and Measures

The study sample consisted of 141 cases of schizophrenia and 142 controls (n = 283). Patients were recruited following consecutive attendance from out-patient clinics at three public mental health services of Granada, Jerez de la Frontera, and Jaén (Andalusia, Spain). Controls were all recruited from different primary care centres from the same catchment areas as patients. All participants were European from South Spain.

Inclusion criteria in the group required patients to have a diagnosis of schizophrenia and less than 5 years since the first episode. All patients had to fulfil the ICD-10 criteria for schizophrenia, as established using the Spanish validated version of the Schedule for Clinical Assessment in Neuropsychiatry (SCAN) [14]. The SCAN was administered by a fully trained research psychologist. Schizophrenia patients were further characterised by the use of the Positive and Negative Symptoms of Schizophrenia Scale (PANSS) [15], and a thorough cognitive test battery. Exclusion criteria in this group of patients were: having a diagnosis of a chronic infectious disease, having received a transplant, presenting CD4+ T-cell counts out of normal range, receiving treatment with immunosuppressive medicines, or suffering from intellectual disability.

The control group inclusion criteria were: no personal or family history of mental disorders or suicide, and not being under pharmacological or psychological treatment. Controls were unrelated subjects who were all screened out for any mental disorder by using of the MINI International Neuropsychiatric Interview [16]. As in the case of the patients, exclusion criteria for the control group were: having a diagnosis of chronic infectious disease, having received a transplant, presenting CD4+ T-cell counts out of normal range, receiving treatment with immunosuppressive medicines, or suffering from intellectual disability.

### 2.2. Microbiological Analyses

A biological sample of blood was obtained from each participant for both microbiological and genetic analyses. Microbiological determinations were performed as follows.

#### 2.2.1. Detection of IgG Anti-*T. gondii* by ELISA

ELISAs were performed using a commercial TOXOPLASMA IgG ELISA kit (Vircell, Granada, Spain) to detect specific IgG antibodies for *T. gondii* in serum following the manufacturer’s instructions. The absorbance of each sample was read with an ELISA plate reader with a 450 nm filter. Test samples with absorbances equal to or greater than the cut-off value were considered to be positive for anti-*T. gondii* IgG.

#### 2.2.2. Detection of *T. gondii* DNA by Nested PCR

In order to obtain further evidence of *T. gondii* parasitation, the presence of *T. gondii* DNA from the peripheral blood was assessed both in cases and controls by using a nested PCR (n-PCR) which had 10 DNA copies/μL analytic sensitivity. In brief, DNA was extracted from the peripheral blood with the ReliaPrep™ Blood gDNA Miniprep System (Promega Biotech Ibérica, Madrid, Spain) according to the manufacturer’s instructions. A 97 bp fragment of B1 gene *T. gondii* was amplified using outer primers 5’-GGAACTGCATCCGTTCATGAG-3’ and 5’-TCTTTAAAGCGTTCGTGGTC-3’, and inner primers 5’-TGCATAGGTTGCAGTCACTG-3′ and 5’-GGCGACCAATCTGCGAATACACC-3’. In both external and internal PCR, the reaction mixtures contained 1× reaction buffer (pH 8.5), 150 µM of each dNTP, 2.5 mM MgCl_2_, 0.4 µM of each oligonucleotide primer, and 1.25 unit of GoTaq^®^ Flexi DNA Polymerase (Promega Biotech Ibérica) in a total volume of 25 µL. For the external PCR, 1 µL of the extracted DNA was added to 24 µL of the PCR reaction mixture. The PCR conditions were detailed elsewhere [17].

### 2.3. Genetic Analyses

DNA was extracted from the blood following standard procedures of DNA precipitation using alcohol and salts. DNA concentration was measured by absorbance using Nanodrop2000 (Thermo Fisher Scientific, Wilmington, NC, USA). Samples were genotyped for the *COMT* Val105/158Met polymorphism (rs4680) using TaqMan^®^ StepOnePlusTM Real-Time PCR System (Applied Biosystems, Foster City, CA, USA) following manufacturer’s instructions. The genetic context sequence was CCAGCGGATGGTGGATTTCGCTGGC[A/G]TGAAGGACAAGGTGTGCATGCCTGA. The validity of each genotypic group or cluster was confirmed by randomly regenotyping 10% of the samples. In all cases, genotypes were confirmed.

### 2.4. Statistical Analyses

The Hardy–Weinberg equilibrium was checked in the entire sample, both in the cases and controls, using the genhw STATA command. Analyses were performed under the aprioristic assumption of an additive genetic model. Pearson’s chi^2^ tests were performed to test for independent associations between: (i) *COMT* Val105/158Met polymorphic variation and schizophrenia, and (ii) *T. gondii* infection and schizophrenia. These main effects were also tested crude, and adjusted for sex and age using logistic regression analyses. Two-way (GxE) interactions on the presence of schizophrenia were examined using logistic regression analyses. Specifically, generalised linear models with binomial distribution were used to estimate the odds ratios (OR) with 95% confidence intervals (CIs). Tests were implemented using the binreg STATA command. In the case of the interaction analysis, a detailed assessment was conducted to compare the different risk effects for schizophrenia conferred by *T. gondii* infection across different genotypes. Analyses were also performed crude, and adjusted for sex and age. Statistical power was calculated by using QUANTO v.1.2.4 software (http://hydra.usc.edu/gxe/ (accessed on 1 May 2015)). According to these calculations, our sample size had 80% power (significance level of α < 0.05) to detect a gene–environment interaction effect of at least 3.7 if we assumed an additive genetic model, a prevalence for schizophrenia of 1%, a 14% prevalence of *T. gondii* infection in the general population according to previous studies [6], and a frequency of the hypothesized risk allele (*COMT* Met158) of 0.43, as reported in Spanish population [18].

## 3. Results

### 3.1. Sociodemographic Sample Characteristics 

The sociodemographic profile of our sample is summarized in Table 1. Patients were predominantly males and significantly younger than the controls. Although the groups did not differ in educational achievement, they exhibited differences regarding marital status.

### 3.2. Main Effect of the COMT Val105/158Met Polymorphism

*COMT* genotype frequencies were in Hardy–Weinberg equilibrium both in patients (χ^2^ = 0.441; df = 2; *p*-value = 0.80) and controls (χ^2^ = 0.545; df = 2; *p*-value = 0.76), supporting the absence of genotyping artefacts. The analysis of the association between this polymorphism and individual groups (patient vs. control) revealed that there were no statistically significant differences in the distribution of allelic and genotypic frequencies (χ^2^ = 0.85; df = 1; *p*-value = 0.35; and χ^2^ = 0.95; df = 2; *p*-value = 0.62; respectively) when comparing schizophrenia patients versus controls (details shown in Table 2). These results remained unaltered after adjusting for sex and age.

### 3.3. Main Effect of T. gondii Infection

We explored the association between schizophrenia and previous *T. gondii* infection as measured by the blood presence of anti-*T. gondii* IgG or parasite DNA. Infection by *T. gondii* determined by positive IgG was significantly more common in cases of schizophrenia than that in the controls (χ^2^ = 11.71; df = 1; *p*-value < 0.001). This association remained robust and significant after adjusting for sex and age. In our sample, *T. gondii* infection was found to increase the risk of schizophrenia by 2.5-fold (OR 2.50 [95% CI 1.47–4.23]; *p*-value = 0.0006; Table 3). No parasite DNA was found in the peripheral blood.

### 3.4. COMT–T. gondii Interaction

**Figure 1:**
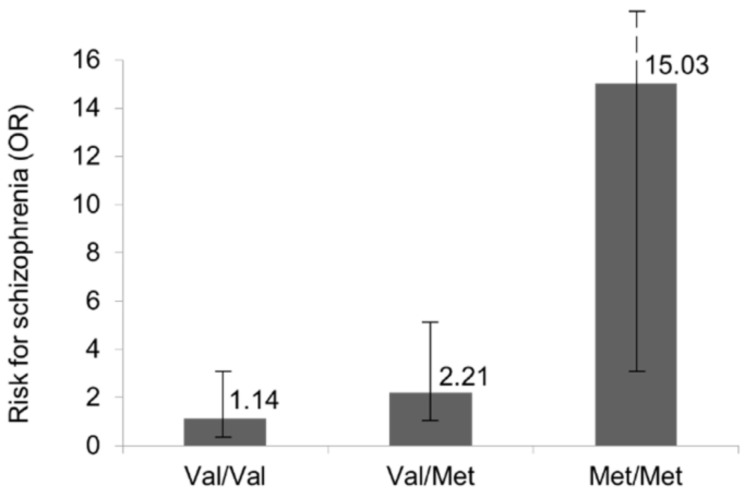
Risk for schizophrenia conferred by *T. gondii* infection across different *COMT* Val105/158Met phenotypes.

Table 4 shows a stratified analysis of the levels of exposure for both *COMT* genotypes and the *T. gondii* infection among cases and controls. In the group of noninfected individuals, no differences in genotypic proportions were found (χ^2^ = 1.15, df = 2, *p*-value = 0.564). In the group of individuals with previous *T. gondii* infection, the genotypic proportions differed significantly between the controls and patients with schizophrenia (χ^2^ = 7.35, df = 2, *p*-value = 0.025). When exploring the interaction between *COMT* and *T. gondii*, we found a statistically significant effect modification by the *COMT* genotype on the risk effect exerted by *T. gondii* infection on schizophrenia (χ^2^ for the interaction = 7.42, *p*-value = 0.0065). Such gene–environment interaction remained significant after adjusting for sex and age (χ^2^ for the interaction = 7.28, *p*-value = 0.007). Thus, *T. gondii* parasitation almost conferred an increased risk for schizophrenia in infected Val/Met subjects (OR 2.21 (95% CI 1–4.93); *p*-value = 0.05) when compared to infected Val/Val individuals (OR 1.14 (95% CI 0.4–3.23); *p*-value = 0.80). This risk was even higher and especially significant in infected subjects carrying the A/A genotype (OR 15.03 (95% CI 2.73–82.64); *p*-value = 0.002) (Figure 1).

## 4. Discussion

The main aim of the present study was to identify the putative interaction between *T. gondii* infection and genetic variation of the *COMT* gene on the risk for schizophrenia. For this purpose, we analysed a sample consisting of patients with schizophrenia and healthy controls in which microbiological determinations for *T. gondii,* and genetic analyses to characterise the functional *COMT* Val105/158Met polymorphism were performed. Our results show that *COMT* variation and exposure to *T. gondii* interact and modify the risk for schizophrenia. Particularly, subjects carrying the A/A genotype were significantly more vulnerable to the risk for schizophrenia conferred by *T. gondii* infection. In these subjects, the deleterious effect of *T. gondii* was significantly increased when compared to infected subjects with other *COMT* Val105/158Met combinations.

To our knowledge, this is the first study analysing the interaction between this specific genetic variation and this environmental infectious risk factor. Due to its role in many neurobiological processes, catechol-O-methyltransferase has been studied for many years. *COMT* is the principal metabolizer of dopamine, and it contributes to regulating the amount of this neurotransmitter in different brain areas. As changes in the dopamine system are thought to play a major role in the development of psychiatric diseases, functional dopamine-associated variations in the 22q11 chromosome, such as *COMT* Val105/158Met, are strong candidates for exploring the genetic components of mental disorders [5]. Such polymorphism, also known as rs4680, is characterised by the fact that it has two possible genetic variants with different functional repercussions on the protein for which they code, with the Val158 allele determining greater activity than the Met158 allele does [4]. The Met158 allele is associated with higher tonic dopamine levels [19]. Studies on the Val105/158Met polymorphism produced conflicting results until now. Excessive dopaminergic activity at the mesolimbic brain areas is currently the most accepted neurochemical substrate in schizophrenia and the base for its most accepted treatment, i.e., antipsychotics which block postsynaptic dopamine receptors [20].

In our sample, we did not find significant differences between patients with schizophrenia and controls in relation to the *COMT* genotype when explored as a single potential risk factor. These results are, among others, in accordance with those found by Rosa et al. [21], and Munafò et al. [22], who also failed to find a significant association between *COMT* genetic variation and schizophrenia. *COMT* might, however, confer a small risk for schizophrenia that is difficult to detect or it may not directly act on the risk for the disease, but modulate the impact of other risk factors such as environmental ones. In fact, despite the high heritability of schizophrenia, it is well-recognized that genetic factors not only coexist with nongenetic risk factors, but also interact with them [9]. Thus, the influence of *COMT* Val105/158Met on behaviour may be better understood in terms of gene–environment interactions, as both type of factors seem to converge on common networks implicated in brain function [23].

In the present study, we also explored the association between infection by *T. gondii* and schizophrenia. Previous studies showed that an excess of infected individuals show psychotic symptoms [24], and the role of *T. gondii* as a risk factor for schizophrenia was repeatedly supported. Regarding the magnitude of the risk, two meta-analyses conducted by our group [6,25] show that, when comparing schizophrenia patients with controls, those exposed to the infection were about 2.7 times more likely to suffer from schizophrenia. A recent meta-analysis of anti-*T. gondii* IgG seropositivity in patients with schizophrenia reinforced these findings [12].

*T. gondii* causes cysts that can be ubiquitous on most brain areas, but tend to be preferentially located in both the amygdala and the nucleus accumbens. The nucleus accumbens and the amygdala are key relay centres in the dopaminergic mesolimbic system whose distortion is, as mentioned, central to schizophrenia, particularly when local excessive dopamine levels are present [26,27]. *T. gondii* is thought to provide excessive levels of tyrosine hydroxylase in the cyst areas, and this enzyme is the rate-limiting factor in dopamine synthesis that becomes increased in such areas [13]. Beyond the dopamine hypothesis, *T. gondii* infection can also alter a variety of other neurotransmitters that have secondary neurochemical roles in schizophrenia, such as serotonin and glutamate, which can be responsible for other behavioural changes included in this complex mental disorder [28,29].

On the other hand, different genomewide approaches support that genetic variation in genes participating in inflammatory processes and infection may modify the risk for psychosis [3,30]. With a gene–environment perspective that combined GWAS and infectious agent’s information, Avramopoulos et al. [31] described HLA-related genes as effect modulators of some infectious agents, including *T. gondii*. Following a candidate gene approach, a recent study on a Lebanese population that also compared the prevalence of anti-*T. gondii* IgG in patients with schizophrenia and controls suggested that genetic variation in a peptidase involved in neuroinflammation could be implicated in the occurrence mechanism of this condition following *T. gondii* infection [32]. Furthermore, the neurotropic infection by *T. gondii* is also related to inflammatory changes in the brain such as the elicitation of a dominant lymphocyte Th1 response involving interferon-γ (IFN-γ), interleukin 12, IL-18, and tumour necrosis factor α (TNF-α). These and other proinflammatory changes are established correlates of schizophrenia, and are implicated in structural and cerebral changes that are common in both schizophrenia and *T. gondii* infection [33]. Thus, both structural [34] and functional [35] neuroimaging studies found smaller and less functional presentations of nucleus accumbens in schizophrenia compared to controls. Similarly, structural and functional amygdala abnormalities are also well-known in schizophrenia [36]. Recent MRI evidence of grey matter reduction in schizophrenia patients positive for *T. gondii* infection indicates that it could be mediated by both the direct effect of associated inflammation processes and an excess of dopamine metabolites, which is inversely correlated with grey matter volume loss [33].

**Figure 2:**
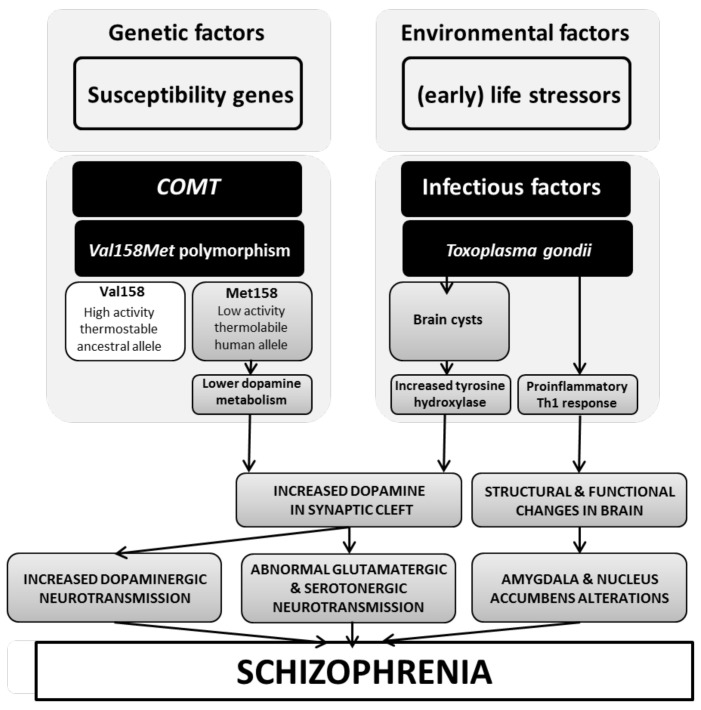
Plausible interaction cascade between *COMT* and *T. gondii* infection in schizophrenia.

Subjects who are carriers of the *COMT* Met158 allele (those with lower levels of *COMT* activity) and particularly Met158 homozygotes (those with the lowest *COMT* functionality) are less efficient in dopamine metabolism and hence liable to have excessive dopaminergic activity [4,37]. Such individuals would be at increased risk for the amplification of such dopamine excess if infected by *T. gondii*, which also increases dopamine levels, particularly in those areas and brain circuits where dopamine excess is related to risk for schizophrenia. Hence, our reported interaction could have a plausible neurobiological explanation in a potential multiplicative effect of these two different channels of dopamine excess that would, in turn, increase the risk of psychosis and schizophrenia. Figure 2 summarizes a potential explanatory causal cascade integrating our findings with the elements discussed above.

### Limitations

Although this study provides ground-breaking results in the field of gene–environment interactions, it has some limitations that should be mentioned. The main limitation of our analyses was not being able to determine the genetic lineage of the parasite and the timing of the infection, which are key factors determining the results of infection with *T. gondii* [38]. Our serological data were based on the detection of IgG, a marker of having been infected, but we could not know the period during which the subjects were infected or the genetic lineage of the parasite. If these data had been available, we could have addressed another type of study, taking into account the different genetic lineage characteristics and whether the infection had been prenatal, during infancy, or in adulthood. If we had been able to discriminate this factor, our results would have probably resulted stressed in the individual’s developmental period where the infections acquire special relevance. It would have been ideal to have been able to test the reported gene by environment interaction using the quantitative and qualitative serum levels of IgG antibodies. Additionally, we did not perform an MRI scan of the participants, which would have facilitated the ascertainment of *T.gondii* infection repercussions, as that was beyond the scope and resources of the study. Finally, we did not assess the clinical indicators of *T.gondii* infection in participants. Nevertheless, in the absence of better evidence, our data support a plausible link between genetic and infectious risk for schizophrenia. We did not screen HIV-1 infection, but none of the participants was receiving treatment with immunosuppressors or presenting an abnormal CD4+ T cell count, which generally becomes impaired in the presence of HIV infection. The sample size was limited but sufficient to detect the reported interaction. Lastly, another possible limitation of our study could be the differences found in sex and age distribution between the cases and controls. However, we took it into account in our analyses, which were all adjusted by these potential confounders.

## 5. Conclusions

The role of certain genetic and infectious factors in the aetiology of schizophrenia seems to be better explained if considered in a synergistic-interaction manner. According to our results, increased risk for schizophrenia could plausibly be exerted via the direct effects of the infection of the brain (circuitry distortion via cyst formation and direct inflammatory factors effects, among others) and/or via neurochemical changes such as increased dopamine mediated by both poor *COMT* activity and increased dopamine synthesis caused by *T. gondii* infection.

## Figures and Tables

**Table 1:** Demographic characteristics of patients with schizophrenia and controls.

Characteristics	Patients N (%)	Controls N (%)	*p*-Value
Mean age (n = 283)	33.29 (SD = 8.58)	39.08 (SD = 10.83)	t = 4.94, *p*-value < 0.001
Sex (n = 283)			
Male	95 (67.4)	67 (47.2)	χ^2^ = 12.76, df = 1, *p*-value < 0.001
Female	46 (32.6)	75 (52.8)	
Education (n = 271)			
Elementary education	21 (16.3)	15 (10.6)	χ^2^ = 7.33, df = 3, *p*-value < 0.062
Junior school	59 (45.7)	60 (42.3)	
High school	44 (34.1)	50 (35.2)	
University degree	5 (3.9)	17 (12)	
Marital status (n = 270)			
Single/never married	97 (75.2)	41 (29.1)	χ^2^ = 61.03, df = 3, *p*-value < 0.001
Living with a partner	21 (16.3)	84 (59.6)	
Divorced/separated	11 (8.5)	15 (10.6)	
Widowed	0 (0)	1 (0.7)	

**Table 2:** *COMT* allelic and genotypic frequencies in patients with schizophrenia and controls.

Allelic and Genotypic Frequencies	Controls N (%)	Patients N (%)
Allelic frequencies		
G	167 (58.80)	155 (54.96)
A	117 (41.20)	127 (45.04)
	χ^2^ = 0.85; df = 1; *p*-value = 0.35	
Genotypic frequencies		
G/G	46 (32.4)	40 (28.4)
G/A	75 (52.8)	75 (53.2)
A/A	21 (14.8)	26 (18.4)
	χ^2^ = 0.95; df = 2; *p*-value = 0.62	

**Table 3:** Frequency of *T. gondii* infection in patients and controls.

Infection by *T. gondii*	Controls (N (%))	Patients (N (%))
Yes	29 (20.42)	55 (39)
No	113 (79.58)	86 (61)
	OR = 2.50; 95% CI: 1.47–4.23; *p*-value = 0.0006	

**Table 4:** Stratified analysis of levels of exposure for both *COMT* genotypes and *T. gondii* infection among cases and controls.

Infection by *T. gondii*	Group	Val/Val	Val/Met	Met/Met
No	Controls	35 (31%)	59 (52.2%)	19 (16.8%)
	Cases	30 (34.9%)	46 (53.5%)	10 (11.6%)
Yes	Controls	11 (37.9%)	16 (55.2%)	2 (6.9%)
	Cases	10 (18.2%)	29 (52.7%)	16 (29.1%)

## Data Availability

Not applicable.

## References

[1] van Os J., Rutten B.P., Myin-Germeys I., Delespaul P., Viechtbauer W., van Zelst C., Bruggeman R., Reininghaus U., Morgan C., European Network of National Networks Studying Gene-Environment Interactions in Schizophrenia (EU-GEI) (2014). Identifying gene-environment interactions in schizophrenia: Contemporary challenges for integrated, large-scale investigations. Schizophr. Bull..

[2] Howes O.D., Kapur S. (2009). The dopamine hypothesis of schizophrenia: Version III--the final common pathway. Schizophr. Bull..

[3] Trubetskoy V., Pardiñas A.F., Qi T., Panagiotaropoulou G., Awasthi S., Bigdeli T.B., Bryois J., Chen C.-Y., Dennison C.A., Hall L.S. (2022). Mapping genomic loci implicates genes and synaptic biology in schizophrenia. Nature.

[4] Lachman H.M., Papolos D.F., Saito T., Yu Y.M., Szumlanski C.L., Weinshilboum R.M. (1996). Human catechol-O-methyltransferase pharmacogenetics: Description of a functional polymorphism and its potential application to neuropsychiatric disorders. Pharmacogenetics.

[5] Hosák L. (2007). Role of the COMT gene Val105/158Met polymorphism in mental disorders: A review. Eur. Psychiatry J. Assoc. Eur. Psychiatr..

[6] Arias I., Sorlozano A., Villegas E., Luna J.D.D., McKenney K., Cervilla J., Gutierrez B., Gutierrez J. (2012). Infectious agents associated with schizophrenia: A meta-analysis. Schizophr. Res..

[7] Shehata A.I., Hassanein F.I., Abdul-Ghani R. (2016). Seroprevalence of Toxoplasma gondii infection among patients with non-schizophrenic neurodevelopmental disorders in Alexandria, Egypt. Acta Trop..

[8] Stilo S.A., Murray R.M. (2019). Non-Genetic Factors in Schizophrenia. Curr. Psychiatry Rep..

[9] Wahbeh M.H., Avramopoulos D. (2021). Gene-Environment Interactions in Schizophrenia: A Literature Review. Genes.

[10] Boksa P. (2008). Maternal infection during pregnancy and schizophrenia. J. Psychiatry Neurosci. JPN.

[11] Torrey E.F., Yolken R.H. (2003). Toxoplasma gondii and schizophrenia. Emerg. Infect. Dis..

[12] Oncu-Oner T., Can S. (2022). Meta-analysis of the relationship between Toxoplasma gondii and schizophrenia. Ann. Parasitol..

[13] Prandovszky E., Gaskell E., Martin H., Dubey J.P., Webster J.P., McConkey G.A. (2011). The neurotropic parasite Toxoplasma gondii increases dopamine metabolism. PLoS ONE..

[14] Wing J.K., Babor T., Brugha T., Burke J., Cooper J.E., Giel R., Jablenski A., Regier D., Sartorius N. (1990). SCAN. Schedules for Clinical Assessment in Neuropsychiatry. Arch. Gen. Psychiatry.

[15] Kay S.R., Fiszbein A., Opler L.A. (1987). The positive and negative syndrome scale (PANSS) for schizophrenia. Schizophr. Bull..

[16] Sheehan D.V., Lecrubier Y., Sheehan K.H., Amorim P., Janavs J., Weiller E., Hergueta T., Balker R., Dunbar G.C. (1998). The Mini-International Neuropsychiatric Interview (M.I.N.I.): The development and validation of a structured diagnostic psychiatric interview for DSM-IV and ICD-10. J. Clin. Psychiatry.

[17] Daniels J.K., Williams N.M., Williams J., Jones L.A., Cardno A.G., Murphy K.C., Spurlock G., Riley B., Scambler P., Asherson P. (1996). No evidence for allelic association between schizophrenia and a polymorphism determining high or low catechol O-methyltransferase activity. Am. J. Psychiatry.

[18] Gutiérrez B., Bertranpetit J., Guillamat R., Vallès V., Arranz M.J., Kerwin R., Fañanás L. (1997). Association analysis of the catechol O-methyltransferase gene and bipolar affective disorder. Am. J. Psychiatry.

[19] Bilder R.M., Volavka J., Lachman H.M., Grace A.A. (2004). The Catechol-O-Methyltransferase Polymorphism: Relations to the Tonic–Phasic Dopamine Hypothesis and Neuropsychiatric Phenotypes. Neuropsychopharmacology.

[20] Jones H.M., Pilowsky L.S. (2002). Dopamine and antipsychotic drug action revisited. Br. J. Psychiatry J. Ment. Sci..

[21] Rosa A., Peralta V., Cuesta M.J., Zarzuela A., Serrano F., Martínez-Larrea A., Fañanás L. (2004). New evidence of association between COMT gene and prefrontal neurocognitive function in healthy individuals from sibling pairs discordant for psychosis. Am. J. Psychiatry.

[22] Munafò M.R., Bowes L., Clark T.G., Flint J. (2005). Lack of association of the COMT (Val158Met) gene and schizophrenia: A meta-analysis of case-control studies. Mol. Psychiatry.

[23] Réthelyi J.M., Benkovits J., Bitter I. (2013). Genes and environments in schizophrenia: The different pieces of a manifold puzzle. Neurosci. Biobehav. Rev..

[24] Yolken R.H., Dickerson F.B., Fuller Torrey E. (2009). Toxoplasma and schizophrenia. Parasite Immunol..

[25] Gutiérrez-Fernández J., Luna Del Castillo J.d.D., Mañanes-González S., Carrillo-Ávila J.A., Gutiérrez B., Cervilla J.A., Sorlozano-Puerto A. (2015). Different presence of Chlamydia pneumoniae, herpes simplex virus type 1, human herpes virus 6, and Toxoplasma gondii in schizophrenia: Meta-analysis and analytical study. Neuropsychiatrc Dis. Treat..

[26] Bird E.D., Spokes E.G., Iversen L.L. (1979). Increased dopamine concentration in limbic areas of brain from patients dying with schizophrenia. Brain J. Neurol..

[27] Weinberger D.R. (1987). Implications of normal brain development for the pathogenesis of schizophrenia. Arch. Gen. Psychiatry.

[28] Webster J.P. (2001). Rats, cats, people and parasites: The impact of latent toxoplasmosis on behaviour. Microbes Infect..

[29] Xiao J., Prandovszky E., Kannan G., Pletnikov M.V., Dickerson F., Severance E.G., Yolken R.H. (2018). Toxoplasma gondii: Biological Parameters of the Connection to Schizophrenia. Schizophr. Bull..

[30] Schizophrenia Working Group of the Psychiatric Genomics Consortium (2014). Biological insights from 108 schizophrenia-associated genetic loci. Nature.

[31] Avramopoulos D., Pearce B.D., McGrath J., Wolyniec P., Wang R., Eckart N., Hatzimanolis A., Goes F.S., Nestadt G., Mulle J. (2015). Infection and Inflammation in Schizophrenia and Bipolar Disorder: A Genome Wide Study for Interactions with Genetic Variation. Potash JB, editor. PLoS ONE..

[32] El Mouhawess A., Hammoud A., Zoghbi M., Hallit S., Haddad C., El Haddad K., el Khoury S., Tannous J., Obeid S., Halabi M.A. (2020). Relationship between Toxoplasma gondii seropositivity and schizophrenia in the Lebanese population: Potential implication of genetic polymorphism of MMP-9. BMC Psychiatry.

[33] Horacek J., Flegr J., Tintera J., Verebova K., Spaniel F., Novak T., Brunovsky M., Bubenikova-Valesova V., Holub D., Palenicek T. (2012). Latent toxoplasmosis reduces gray matter density in schizophrenia but not in controls: Voxel-based-morphometry (VBM) study. World J. Biol. Psychiatry.

[34] Mamah D., Wang L., Barch D., de Erausquin G.A., Gado M., Csernansky J.G. (2007). Structural analysis of the basal ganglia in schizophrenia. Schizophr. Res..

[35] Shergill S.S., Bullmore E., Simmons A., Murray R., McGuire P. (2000). Functional anatomy of auditory verbal imagery in schizophrenic patients with auditory hallucinations. Am. J. Psychiatry.

[36] Lawrie S.M., Whalley H.C., Job D.E., Johnstone E.C. (2003). Structural and functional abnormalities of the amygdala in schizophrenia. Ann. N. Y. Acad. Sci..

[37] Lotta T., Vidgren J., Tilgmann C., Ulmanen I., Melén K., Julkunen I., Taskinen J. (1995). Kinetics of human soluble and membrane-bound catechol O-methyltransferase: A revised mechanism and description of the thermolabile variant of the enzyme. Biochemistry.

[38] Torrey E.F., Yolken R.H. (2019). Schizophrenia as a pseudogenetic disease: A call for more gene-environmental studies. Psychiatry Res..

